# Carbon black suppresses the osteogenesis of mesenchymal stem cells: the role of mitochondria

**DOI:** 10.1186/s12989-018-0253-5

**Published:** 2018-04-12

**Authors:** Yulai Shen, Lu Wu, Dongdong Qin, Yankai Xia, Zhu Zhou, Xuemei Zhang, Xin Wu

**Affiliations:** 1 State Key Laboratory of Reproductive Medicine (SKLRM) & Key Laboratory of Modern Toxicology of Ministry of Education, Nanjing Medical University, Nanjing, 211100 Jiangsu China; 2 Department of Pharmaceutics and Medicinal Chemistry, University of the Pacific, Stockton, 95211 USA

**Keywords:** Mesenchymal stem cells, Osteogenesis, Carbon black, Mitochondrial biogenesis, Mitochondrial dynamics, Mitophagy

## Abstract

**Background:**

The rapid increase in carbon black poses threats to human health. We evaluated the effect of CB (Printex 90) on the osteogenesis of bone-marrow-derived mesenchymal stem cells (MSCs). Mitochondria play an important role in the osteogenesis of MSCs and are potential targets of nanomaterials, so we studied the role of mitochondria in the CB Printex 90-induced effects on osteogenesis.

**Results:**

Low doses of Printex 90 (3 ng/mL and 30 ng/mL) that did not cause deleterious effects on MSCs’ viability significantly inhibited osteogenesis of MSCs. Printex 90 caused down-regulation of osteoblastic markers, reduced activity of alkaline phosphatase (ALP), and poor mineralization of osteogenically induced MSCs. Cellular ATP production was decreased, mitochondrial respiration was impaired with reduced expression of ATPase, and the mitochondrial membrane was depolarized. The quantity and quality of mitochondria are tightly controlled by mitochondrial biogenesis, mitochondrial dynamics and mitophagy. The transcriptional co-activator and transcription factors for mitochondrial biogenesis, PGC-1α, Nrf1 and TFAM, were suppressed by Printex 90 treatment, suggesting that decreased biogenesis was caused by Printex 90 treatment during osteogenesis. Mitochondrial fusion and fission were significantly inhibited by Printex 90 treatment. PINK1 accumulated in Printex 90-treated cells, and more Parkin was recruited to mitochondria, indicating that mitophagy increased to remove the damaged mitochondria.

**Conclusions:**

This is the first report of the inhibitory effects of CB on the osteogenesis of MSCs and the involvement of mitochondria in CB Printex 90-induced suppression of MSC osteogenesis.

**Electronic supplementary material:**

The online version of this article (10.1186/s12989-018-0253-5) contains supplementary material, which is available to authorized users.

## Background

Carbon black (CB) is a fine black powder of nearly pure elemental carbon. It is a manufactured product that has been used for over a century. Commercially available grades of carbon black differ in particle size, morphology, surface area, and structure [1]. CB is one of the top 50 industrial chemicals worldwide, and megatons are produced annually. However, epidemiological studies have indicated a higher frequency of lung cancers, inflammatory responses, nonmalignant respiratory diseases, and cardiac mortality in CB production workers [2–6]. Nanoscale CB particles are responsible for lung function reduction and pro-inflammatory cytokine secretion in CB workers [7].

CB has been used as a representative carbonaceous particle toxicant in air pollution studies [8]. Potential health effects of nano-sized CB particles have been studied in vitro and in vivo. CB is responsible for a number of disorders, including early pulmonary response [9], vascular effects [10], genotoxicity, and reproductive toxicity [11, 12]. CB could act as the vehicles for metal delivery and generate synergistic lung toxicity though autophagy and lysosomal dysfunction [13]. Although the impacts of CB on many biological systems have been studied, its effects on bone health remain unclear. Since exposure to elevated levels of CB is a growing problem in many regions of the world, an understanding of the effect of CB on bone health would be useful.

Bone is a dynamic tissue with constant remodeling coordinated by the activities of osteoblasts and osteoclasts [14]. During bone remodeling, osteoclasts resorb bone, followed by recruitment of bone marrow mesenchymal stem cells (MSCs) for subsequent differentiation and bone formation [15]. MSCs are multipotent stem cells with the potential for self-renewal and differentiation. MSCs can be isolated from many tissues and have the capacity to differentiate into multiple cell lineages in vitro, including osteoblasts [16]. The advantages of using a MSC cell culture system to assess the toxicity of Printex 90 instead of traditional animal experiments include reduced labor, time, and cost. In addition, immortalized cell lines can have abnormal characteristics after transformation [17]. Therefore, primary MSCs is a more suitable cell model to study bone formation.

The differentiation of MSCs requires increased energy, and mitochondria play an important role in the differentiation process. Healthy mitochondria are maintained by mitochondrial biogenesis as well as dynamic fusion and fission events, while damaged mitochondria are segregated and removed through fission and mitophagy [18, 19]. A mitochondrion is unique compared to other organelles as it has its own genome**.** Mitochondrial biogenesis (generation of new mitochondria) occurs from the growth and division of pre-existing mitochondria and is coordinated by both the mitochondrial and nuclear genomes. The most studied molecules involved in mitochondrial biogenesis are nuclear respiratory factors 1 and 2 (Nrf1 and Nrf2), peroxisome proliferator-activated receptor-*γ* co-activator 1 alpha (PGC-1*α*), and mitochondrial transcription factor A (TFAM) [20, 21]. Mitochondria are highly dynamic and constantly undergo fission and fusion. Three proteins, including mitofusin-1 (Mfn1), mitofusin-2 (Mfn2), and optic atrophy protein 1 (Opa1), are involved in the regulation of mitochondrial fusion, and the dynamin-related protein 1 (Drp1) and mitochondrial fission protein 1 (Fis1) control mitochondrial fission [22]. Under stress, the mitochondrial membrane potential (ΔΨ_m_) collapses. Damaged mitochondria are then targeted and removed by mitophagy, the selective autophagy of mitochondria [23]. Many studies have demonstrated that during osteogenic differentiation of MSCs, mitochondrial events such as biogenesis, fusion and fission, and mitophagy are altered [24–29].

Due to their structure and vital functions, mitochondria are key intracellular targets for nanomaterials. Many nanoparticles and nanomaterials can cause mitochondrial dysfunction as well as impaired mitochondrial biogenesis and dynamics [30–34]. This suggests that specific mitochondrial markers may be useful biomarkers for the toxicity assessment of nanomaterials.

We used bone-marrow-derived MSCs to evaluate the effects of a commercial CB nanoparticle, Printex 90, on osteogenesis. Mitochondrial biogenesis and dynamics and mitophagy were studied to explain the mechanisms of the effects of Printex 90 on osteogenesis.

## Methods

### Isolation, culture, and differentiation of bone marrow MSCs

Primary bone marrow mesenchymal stem cells (BM-MSCs) were harvested from 21 d Sprague-Dawley (SD) rats following a previously described protocol with some modifications [35]. Briefly, bone marrow was flushed out from the femoral and tibial bones of rats using 5 mL syringes. Cells were collected in Modified Eagle medium-Alpha (α-MEM)(Corning Cellgro, USA) supplemented with 10% fetal bovine serum (FBS)(Gibco, USA), 1% L-glutamine (Gibco, USA), 1% penicillin-streptomycin (Gibco, USA) and 1% HEPES (Gibco, USA), and then cultured at 37 °C in an incubator with 5% CO_2_. Non-adherent cells were removed by replacing the MSC culture medium every other day. After 5 d of initial culture, the MSCs were trypsinized and passaged for further culture. BM-MSCs from passages 3–5 were used in the study.

MSCs were induced to differentiate into osteoblasts in an osteogenic induction medium of Dulbecco’s modified Eagle medium-High glucose (DMEM-HG)(Hyclone, USA) supplemented with 10% FBS, 10 nmol/L dexamethasone (Sigma Aldrich, USA), 10 mmol/L β-glycerol phosphate (Sigma Aldrich, USA), 50 μg/mL ascorbic acid (Sigma Aldrich, USA), 1% L-glucose, 1% penicillin-streptomycin and 1% HEPES.

Adipogenic differentiation of MSCs was induced in the DMEM-HG medium supplemented with 10% FBS, 1 μmol/L dexamethasone, 100 μmol/L indomethacin (Sigma Aldrich, USA), 0.5 mmol/L 3-isobutyl-1-methylxanthine (IBMX)(Sigma Aldrich, USA), 5 μg/mL insulin (Sigma Aldrich, USA), 1% L-glucose, 1% penicillin-streptomycin and 1% HEPES.

For CB exposure during osteogenic differentiation, Printex 90 at doses of 0, 3 and 30 ng/ml was directly supplemented into the osteogenic induction medium before replacing the regular MSC culture medium, once MSCs in the culture plates reached 60% confluence.

### Cell surface marker expression of MSCs

Expression of surface markers was assessed by flow cytometry using the following conjugated monoclonal antibodies: FITC hamster anti-rat CD29, FITC mouse anti-rat CD44H, FITC mouse anti-rat CD45, FITC mouse anti-rat CD90 (BD Biosciences, San José, CA). MSCs at passage 3 were suspended in PBS and incubated with each antibody at a concentration of 0.5 μg/mL, with unstained MSCs and isotype-control as control.

### Characterization of CB Printex 90

Printex 90 (14 nm primary particle size, purchased from Orion Engineered Carbons, USA) was dispersed in different solvents, including ddH_2_O, PBS and the culture medium, to make a final concentration of 1 mg/mL. All samples were sonicated for 30 min before the test. The particle sizes and zeta potentials of CB Printex 90 in different solvents were assessed by Zetasizer Nano series model ZS (Brookhaven Instruments Co, USA). An amount of 30 μg/mL Printex 90, dispersed in PBS, was used to obtain transmission electron microscope (TEM) images (JEM-200CX,JEOL,JAPAN).

### Cell viability assay

Cell viability was determined by the Cell Counting Kit 8 (CCK-8) (Beyotime Institute of Biotechnology, China) following product instructions. MSCs were seeded in a 96-well plate at a density of 9 × 103 cells per well with 100 μL of culture medium. At 24 h after plating, MSCs were exposed to Printex 90 at concentrations of 0, 0.003, 0.01, 0.03, 0.1, 0.3, 1, 3, 10, 30 μg/mL for 24 h. Ten microliter CCK-8 reagent, diluted with 90 μL medium, was added and reacted at 37 °C for 2 h. After centrifugation at 1500 g for 2 min, the absorbance of reaction supernatant was measured by Infinite M200 Pro (TECAN, Switzerland) at 450 nm. Each assay was repeated at least three times independently. Additionally, the viability of MSCs exposed to Printex 90 at concentrations of 0, 3, 30 ng/mL in osteo-induction media for 7 d was further detected by CCK-8.

### Cellular uptake assay

MSCs were seeded in 24-well plates at a density of 7 × 10^4^ cells/well. At 24 h after plating, MSCs were exposed to Printex 90 at concentrations of 0, 10 μg/mL for 24 h. After trypsinization, a cell pellet was fixed with 2% glutaraldehyde. Cells were imbedded, cut into ultrathin slices, and viewed using a TEM (FEI Tecnai G2 Spirit Bio TWIN, USA).

### Alkaline phosphatase (ALP) staining

Leukocyte Alkaline Phosphatase Kit (Sigma Aldrich, USA) was used for ALP staining according to manufacturer instructions. MSCs were seeded in 24-well plates at a density of 7 × 10^4^ cells/well in culture medium. Until the density of cells reached 60%, MSCs were exposed to Printex 90 at concentrations of 0, 3, 30 ng/mL and induced to differentiate into osteoblasts by osteogenic induction medium. After 10 d induction, the cells were fixed with 4% formaldehyde and 5% citrate in acetone at room temperature for 30 s. The fixed cells were washed with PBS and incubated with 0.2% naphthol AS-BI and 0.2% diazonium salt at room temperature for 15 min. After discarding the working solution and washing the plates with PBS, images were made at 4× magnification using an optical microscope (Nikon, Japan).

### ALP activity assay

ALP activity was determined by the Sensolyte® pNPP Alkaline Phosphatase Assay Kit (Anaspec, USA) according to manufacturer instructions. MSCs were seeded in 96-well plates at a density of 1 × 10^4^ cells/well. At 24 h after plating, cells were exposed to Printex 90 at concentrations of 0, 3, 30 ng/mL and osteogenic differentiation was induced as described above. Cells in each well were washed twice with assay buffer, lysed with Triton-X-100 and collected in a microcentrifuge tube. After incubation at 4 °C for 10 min under agitation, cells were centrifuged at 2500 g for 10 min to collect the supernatant. The supernatant was incubated with p-nitrophenyl phosphate (pNPP) substrate solution and the absorbance was measured using Infinite M200Pro (TECAN, Switzerland) at 405 nm. A standard curve was drawn by using ALP standard solution to determine the concentration of ALP. The protein concentration in each well was measured using BCA Protein Assay Kit (Beyotime Institute of Biotechnology, China) to normalize the relative ALP activity.

### Alizarin red S (ARS) staining

MSCs were seeded in 24-well plates at a density of 7 × 10^4^ cells/well. Cells were exposed to Printex 90 at concentrations of 0, 3, 30 ng/mL and osteogenic differentiation was induced for 21 d. The cells were then washed with PBS, fixed with 10% formaldehyde at room temperature for 10 min and incubated with 40 mmol/L Alizarin Red S (Sigma Aldrich, USA) solution at room temperature for 20 min with shaking. After discarding the working solutions and washing the plates with PBS 4 times, the images were made at 4× magnification using an optical microscope (Nikon, Japan).

### Oil red O staining

MSCs were seeded in 24-well plates at a density of 7 × 10^4^ cells/well and adipogenesis was induced for 14 d. The culture was then fixed with 4% formalin solution for 15 min and subjected to 0.5% Oil Red O (Sigma Aldrich, USA) incubation for 40 min. After discarding the working solutions and washing the plates with 60% isopropanol, images were made at 10× magnification using an optical microscope (Nikon, Japan).

### Intracellular adenosine 5′-triphosphate (ATP) content

The intracellular ATP level was determined with an ATP Assay Kit (Beyotime Institute of Biotechnology, China) according to manufacturer instructions. MSCs were exposed to Printex 90 at concentrations of 0, 3, 30 ng/mL and osteogenic differentiation was induced for 7 d. The cells were then washed twice with PBS, lysed, and collected in microcentrifuge tubes. The cell suspensions were centrifuged at 12000 g for 5 min in 4 °C to collect the supernatant. Fifty microliters of ATP detection working solution, together with 10 μL of the supernatant was added to a black 96-well plate for luminescence analysis using a Centro LB 960 (Berthold Technologies, Germany). A standard curve was drawn using ATP standard solution to determine the concentration of ATP. The protein concentration in each well was measured using BCA Protein Assay Kit (Beyotime Institute of Biotechnology, China) to normalize the relative ATP content.

### Mitochondrial membrane potential (ΔΨ_m_)

After induction, MSCs were incubated with 1:500 diluted JC-10 (Ex = 488 nm; Em = 530 nm) (Keygen Biotech, China) at 37 °C for 30 min and washed with PBS two times. After cells were trypsin-dissociated and collected, red and green fluorescence values were determined using a flow cytometer (BD Biosciences, USA) according to manufacturer instructions.

### The extracellular flux (XF) cell mitochondria stress test

Mitochondrial stress was determined by a XF-96 Flux Analyzer (Seahorse XF96, Seahorse Bioscience, USA) measuring the oxygen consumption rate (OCR) of cells. MSCs were seeded in XF 96-well cell culture plate (Seahorse Bioscience, USA) at a density of 5× 10^3^ cells/well. At 24 h after plating, cells were treated by Printex 90 at the indicated concentration (0, 3, 30 ng/mL) and cells were induced to differentiate to osteogenesis. In this study, three mitochondrial inhibitors were sequentially injected: oligomycin (1 μmol/L), a ATP synthase blocker; then carbonyl cyanide 4-(trifluoromethoxy)phenylhydrazone (FCCP) (1.8 μmol/L), an electron transport chain accelerator; then a mixture of antimycin A (0.5 μmol/L) and rotenone (0.5 μmol/L), the inhibitors of mitochondrial respiratory chain complex I and III, respectively, to shut down the mitochondria oxygen consumption. Several mitochondria stress parameters were measured after injections of these inhibitors, including basal respiration, ATP-linked respiration, proton leak, maximal respiration, spare respiratory capacity and non-mitochondrial respiration [36, 37].

### Relative mtDNA copy number

Relative mtDNA copy number was determined by polymerase chain reaction (PCR) using a ABI 7900HT (Applied Biosystems, USA). Total DNA from treated cells was extracted by Universal Genomic DNA Extraction Kit Ver5.0 (TaKaRa, Japan) according to manufacturer instructions. Then, 16 ng of DNA was applied to PCR using the SYBR Green Ex Taqkit (Takara, Japan) according to manufacturer instructions. The gene expression of mtDNA-encoded cytochrome c oxidase I (mt-COX1) and NADH dehydrogenase subunit 1 (mt-ND1) gene were amplified to determine the Relative mtDNA copy number, with the nuclear-encoded glyceraldehyde-3-phosphate dehydrogenase (GAPDH) gene normalized (primer sequences are listed in Additional file 1: Table S1).

### Quantitative real-time PCR

Total RNA was extracted from cells using TRIzol reagent (Invitrogen Life Technologies Co, USA) according to manufacturer instructions. The concentration of total RNA was measured by NanoDrop 2000 (Thermo Fisher Scientific, USA). One microgram of total RNA was reverse transcribed into cDNA using the Prime Script™ RT Reagent Kit with gDNA Eraser (Perfect Real Time, Takara, Japan). The genomic DNA was removed under 42 °C for 2 min and cDNA was synthesized under 37 °C for 15 min and 85 °C for 30 s. The qRT-PCR was performed on an ABI7900 Fast Real-Time System (Applied Bio systems, USA) using a SYBR Premix Ex Taq™ Kit (Takara, Japan). Data analysis was done by using the comparative Ct method with GAPDH as the normalization control. All the primers for PCR are shown in Additional file 1: Table S1.

### Mitochondria isolation

Mitochondria were isolated from MSCs using the Cell Mitochondria Isolation Kit (Beyotime Institute of Biotechnology, China) according to manufacturer instructions. MSCs were seeded in 10 cm dishes at a density of 1.2 × 10^6^ cells/dish, exposed to Printex 90 at concentrations of 0, 3, 30 ng/mL and osteo-induced for 7 d. After trypsinization and washing with PBS, cells were suspended and homogenized with extraction buffer, and then centrifuged at 600 g for 10 min at 4 °C. The supernatant was additionally centrifuged at 11000 g for 10 min to collect the final pellet, which consisted of mitochondrial protein. The concentration of mitochondrial protein was measured using a BCA Protein Assay Kit (Beyotime Institute of Biotechnology, China) for further study.

### Western blot

Whole-cell proteins were lysed by RIPA (Beyotime Institute of Biotechnology, China) on ice and collected by centrifugation at 12,000 rpm for 15 mins. Protein concentrations were quantified by the BCA Protein Assay Kit (Beyotime Institute of Biotechnology, China). Total protein (30 μg) of each sample was separated on a 10% sodium dodecyl sulfate (SDS)-polyacrylamide gel electrophoresis and transferred to polyvinylidene fluoride (PVDF) membranes (Millipore, USA). After the membranes were blocked in TBST containing 5% nonfat milk for 1 h, the blots were incubated with primary antibody overnight at 4 °C. After washing three times (5 mins each) in TBST buffer, the membranes were incubated with horseradish peroxidase (HRP)-conjugated secondary antibody. The immunoreactive bands were visualized using chemiluminescence using the ECL Western blotting detection kit (Advansta, USA) according to manufacturer instructions. The primary antibodies used in this study included mouse anti-GAPDH (Beyotime Institute of Biotechnology, China) (1:1000), mouse anti-total OXPHOS (Abcam, USA) (1:1000), mouse anti-TFAM (NorvsBiologicals, USA)(1:1000), rabbit anti-RUNX2 (Cell Signaling Technology, USA) (1:1000), rabbit anti-PGC-1alpha (Proteintech, USA) (1:250), rabbit anti-NRF1 (Abcam, USA) (1:1000), rabbit anti-MFN2 (Abcam, USA) (1:1000), rabbit anti-DRP1 (Abcam, USA) (1:1000), rabbit anti-PINK1 (Abcam, USA) (1:1000) and rabbit anti-Parkin (Abcam, USA) (1:1000). The HRP-conjugated goat anti-mouse and goat anti-rabbit secondary antibodies were both purchased from Jackson ImmunoResearch (USA) (1:8000). Grey-scale values of each band indicating protein were quantified and normalized by GAPDH.

### Statistical analysis

All data are presented as mean ± standard deviation (SD). Graphpad 5.0 was applied for the statistical analysis. The experiments were repeated in triplicate (*n* = 3). Statistical analysis was performed using One-way ANOVA to evaluate the significance of the experimental data. The data are indicated with (*) for *p* < 0.05, (**) for *p* < 0.01 and (***) for *p* < 0.001.

## Results and discussion

### Printex 90 characterization

**Fig. 1:**
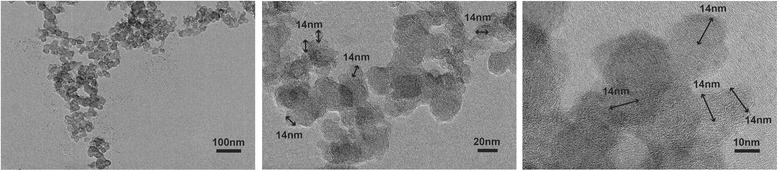
TEM images of Printex 90. The primary particle size is close to 14 nm. From left to right, scale bar = 100 nm, 20 nm and 10 nm

CB (Printex 90) was characterized by evaluating its morphology, size, hydrodynamic diameter and zeta potential (Fig. 1). When dispersed in PBS, Printex 90 tended to aggregate (Fig. 1). Under TEM Printex 90 was spherically shaped with a narrow size distribution. The primary particle size was 14 nm (Fig. 1), which was consistent with manufacturer information. Dynamic light scattering analysis showed that Printex 90 dispersed as small aggregates in culture medium with 10% FBS, compared with the sizes of dispersions in PBS and water (Additional file 2: Figure S1a-b). The zeta potential of Printex 90 depended on the solvent, which could be attributable to the interactions of Printex 90 with ions, proteins or other biomacromolecular components in the dispersed solutions [38].

### Identification of rBM-MSCs

**Fig. 2:**
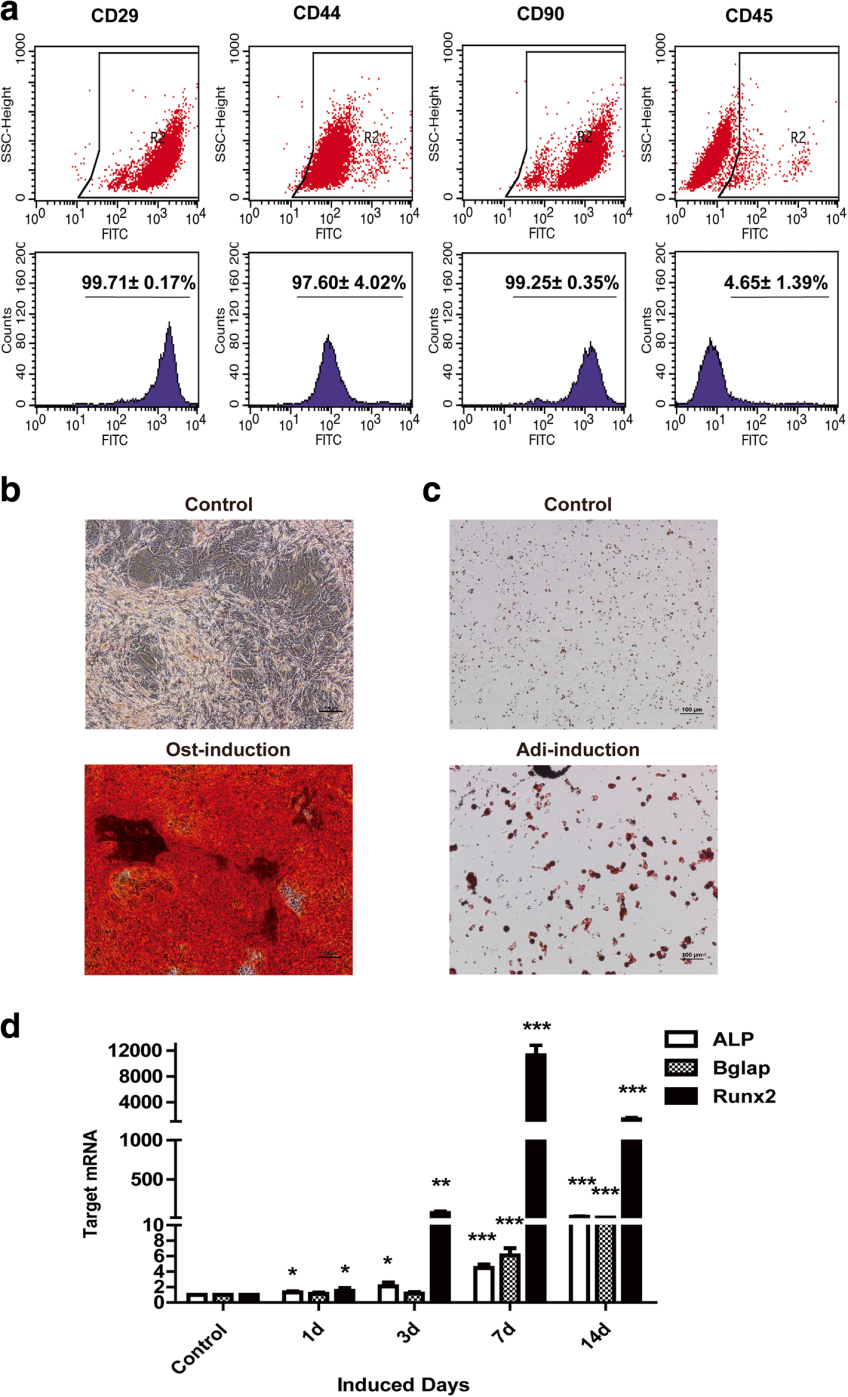
Characterization of mesenchymal stem cells (MSCs) isolated from rat bone marrow. **a** Flow cytometry was used to quantify the cell surface markers of rat MSCs. **b** Alizarin Red S staining was used to show the mineralization nodules after osteogenic induction for 21 d. MSCs were cultured and induced by osteogenic induction media for 21 d. Cells were fixed by 10% formaldehyde and stained with Alizarin Red S and imaged were collected in × 10 magnification with a light microscope. Bar = 100 μm. **c** Oil red O staining showed the lipid droplets after adipogenic induction for 14 d. MSCs were fixed by 4% paraformaldehyde solution and stained with Oil red O and images were collected in × 10 magnification with the light microscope. Bar = 100 μm. **d** RT-PCR demonstrated the up-regulation of osteoblast marker genes, Alp, Runx2 and Bglap, after osteo-induction for different days. MSCs were cultured and induced by osteogenic induction media for indicated days. Then mRNA was collected by TRIzol and reverse transcribed by a reverse transcription kit, and PCR was performed with a SYBR green kit (see methods). Experiments were repeated three times independently

Primary rBM-MSCs were isolated from rat bone marrow and successfully passaged. MSCs adhered to the plastic culture dishes and demonstrated a typical small spindle-shaped morphology. Immunophenotypic characterization was performed by flow cytometry. The isolated cells were positive for mesenchymal-associated markers, such as CD29 (99.71 ± 0.17%), CD44 (97.60 ± 4.02%), and CD90 (99.25 ± 0.35%), with only a small percent of CD45 positive hematopoietic cells (Fig. 2a).

Another key property of MSCs is that they could be readily induced to differentiate. After 21 d of osteogenic induction, we observed a large number of mineralized nodules, revealed by Alizarin red S staining (Fig. 2b). After 14 d of adipogenic induction, a significant number of lipid droplets were detected by oil red O staining (Fig. 2c), confirming that primary MSCs have a high potential ability to differentiate. During osteogenic differentiation, many osteogenic marker genes are transcribed. Runt-related transcription factor 2 (Runx2) is the master transcription factor and it regulates the transcription of another osteogenic marker gene, osteocalcin (Ocn, Bglap), involved in controlling the mineralization process at a later stage [39]. ALP is another marker of osteogenic differentiation and was highly expressed at early stages (7–10 d) [40]. To assess the expression profile of osteogenic marker genes during the osteogenic differentiation of MSCs, MSCs were osteogenically induced from 0 to 14 d. At 24 h after osteo-induction, the mRNA transcripts, Runx2, Bglap and ALP, were not different from control while at 7 d or 14 d post induction, all were significantly up-regulated (Fig. 2d), suggesting the progression of MSCs into a more differentiated stage as mature osteoblasts. Since osteogenic marker genes were up-regulated 7 d after osteogenic induction, we chose 7 d post osteogenic induction in experiments to evaluate Printex 90 effects on osteogenesis.

### Printex 90 affects the viability of MSCs in a dose-dependent manner

**Fig. 3:**
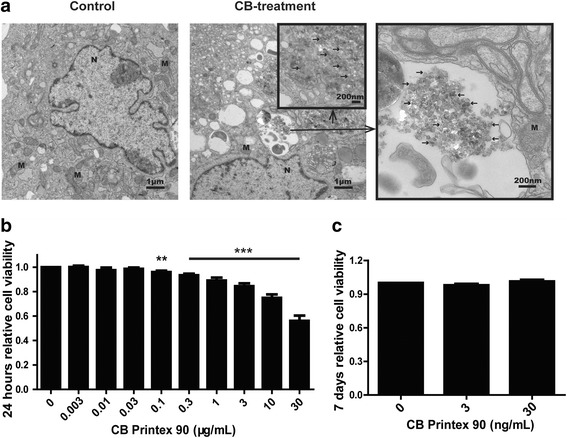
Effects of Printex 90 on MSCs viability. **a** TEM images of MSCs uptaking Printex 90. Untreated cells (Control) showed no Printex 90 inside of cells whereas Printex 90-treated MSCs (CB- treatment) showed the presence of Printex 90 (shown at the arrow) in the cytoplasm but no in the nuclear. N: nucleus; M: mitochondria. Scale bar in normal graphs = 1 μm and in graphs with black box =200 nm. MSCs were treated with Printex 90 at indicated concentration for 24 h (**b**) or 7 d (**c**). After treatment, CCK-8 reagent was added to the culture and incubated for another 2 h and the absorbance was recorded at 450 nm

Cellular uptake of Printex 90 was the initial step leading to a cytotoxic response. Therefore, TEM was used to evaluate whether MSCs were able to uptake Printex 90. After cells were exposed to 10 μg/mL Printex 90 for 24 h, we observed the presence of Printex 90 in MSCs. No Printex 90 was seen inside the nucleus or the nuclear membrane of exposed cells (Fig. 3a).

After confirming the uptake of Printex 90 by MSCs, we studied the cytotoxicity of Printex 90 on MSCs using the CCK-8 assay kit. WST-8 substrate provided in the kit was reduced to formazan dye by dehydrogenases in the cells. To avoid the potential interference of carbon nanoparticles with CCK-8, a control test indicated no difference of the absorbance between Printex 90 supplemented medium and normal medium (Additional file 3: Figure S2). Therefore, the absorbance of formazan dye is directly proportional to the number of living cells. MSCs were exposed to Printex 90 with the indicated concentrations for 24 h. As shown in Fig. 3b, a Printex 90 dosage of 0.03 μg/mL or below had no obvious detrimental effect on cell viability. However, cell viability was significantly decreased with Printex 90 dosage and the decrease was dose-dependent. Since we evaluated Printex 90 effects on osteogenic differentiation for 7 d, we analyzed the cell viability after incubation with 3 ng/mL or 30 ng/mL Printex 90 in osteo-induction media for 7 d. Neither dosage of Printex 90 had significant effects on cell viability (Fig. 3c). Therefore, we selected the 3 ng/mL and 30 ng/mL dosages for further studies.

### The effects of Printex 90 on the osteogenesis of MSCs

**Fig. 4:**
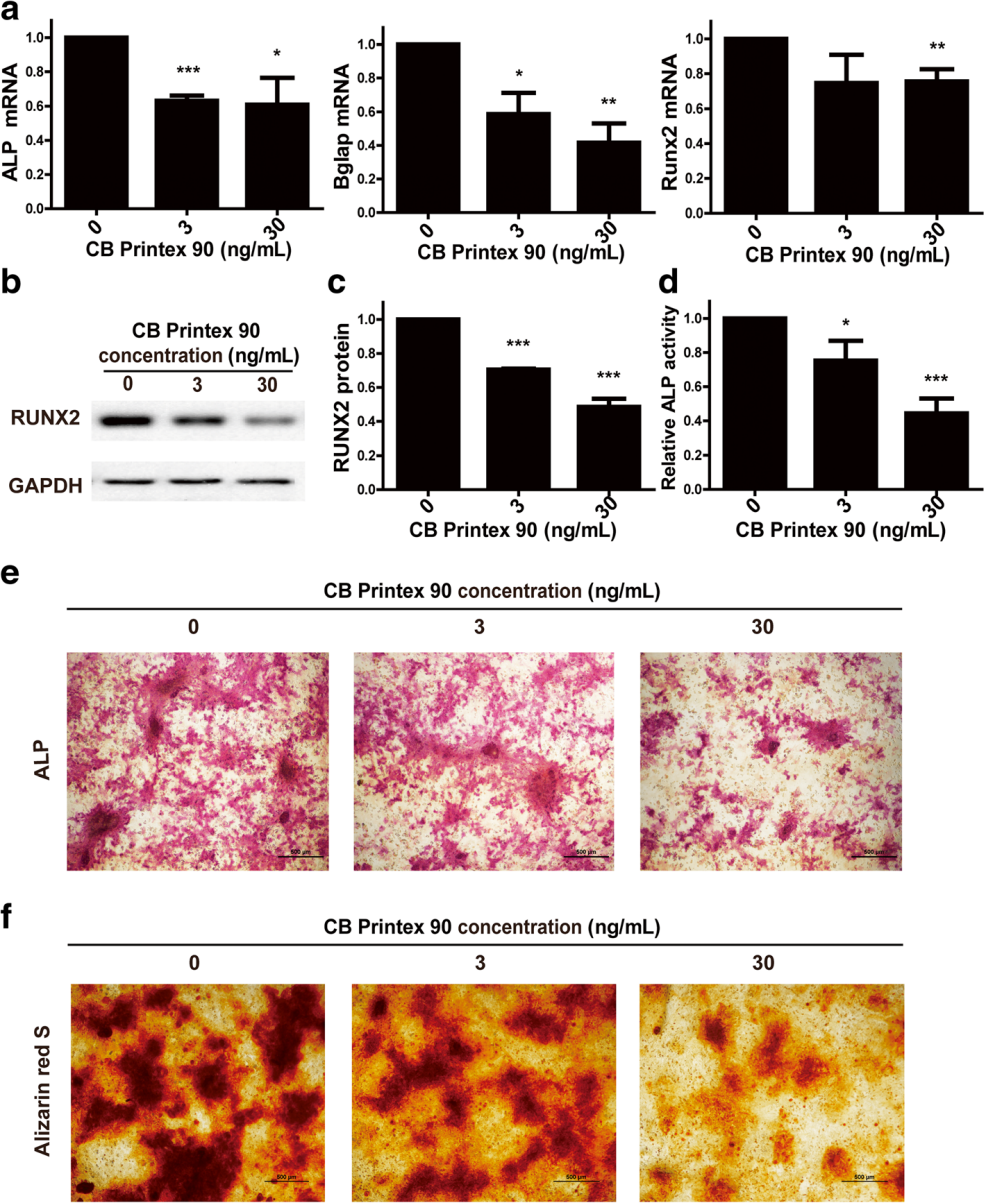
Effects of Printex 90 on osteogenesis of MSCs. MSCs were treated by Printex 90 at indicated concentration and induced to osteogenic differentiation for 7 d. After treatment, mRNA and proteins were collected for RT-PCR analysis (**a**) and Western blot (**b**), respectively. **c** The quantification of Western blot. Printex 90 treated cells were induced to osteogenic differentiation for 10 d followed by ALP activity (**d**) or ALP staining (**e**). (**f**) Alizarin Red S staining showed the mineralization nodules after osteogenic induction for 21 d. Printex 90 treated cells were induced to osteogenic differentiation for 21 d. Cells were fixed and stained by Alizarin Red S. Images were collected with 10× magnification of the light microscopy. Bar = 500 μm

For osteogenic differentiation, Printex 90 was directly supplemented into osteogenic induction medium before replacing the regular culture medium when MSCs seeded into the culture plates reached 60% confluence. MSCs were osteogenically induced for 7 d with 0, 3 or 30 ng/mL Printex 90 treatment which had previously exhibited no deleterious effects on MSCs viability. RT-PCR revealed that Printex 90 treatment resulted in a significant down-regulation of osteogenic marker genes, including ALP, Bglap and Runx2 (Fig. 4a). Western blot assay demonstrated that both 3 and 30 ng/mL Printex 90 significantly suppressed RUNX2 protein expression (*p* < 0.05), 0.70 ± 0.01and 0.49 ± 0.05 respectively (Fig. 4b-c). ALP activity is correlated with matrix formation in osteoblasts. Therefore, we compared the ALP activity between the control and Printex 90-treated cells after 7 d osteo-induction. The Printex 90 inhibited ALP activity in a dose dependent manner (Fig. 4d). The staining in Printex 90-treated MSCs was faint compared to the control after 10 d of osteo-induction (Fig. 4e). Effects of Printex 90 on the mineralization potential were revealed by Alizarin red S staining [41]. Mineralized nodules are typically observed at terminal differentiation and thus MSCs were osteo-induced for 21 d. Figure 4f shows numerous dark brown colored nodules in control cells while both 3 and 30 ng/mL Printex 90 treated cells formed reduced numbers of relatively smaller nodules.

These data demonstrate that Printex 90 can suppress the osteogenic differentiation of MSCs. This conclusion was supported by the down-regulation of Runx2, ALP and Bglap, suppressed RUNX2 protein expression, reduction of ALP activity, and the formation of smaller and less mineralized nodules. Other studies have shown that carbon-based nanoparticles, either commercially available or formed in bread-making process, have moderate toxic effects on self-renewal of MSCs. However, these studies did not investigate nanoparticle effects on osteogenic differentiation [42, 43]. Studies on the purple sea urchin (*Paracentrotus lividus*) have demonstrated that the exposure of sea urchin sperm to CB impaired primary mesenchymal cell migration and anomalous arrangements of the skeletal rod [44]. There are no similar reports involving mammalian systems. Our present work is the first to demonstrate the inhibitory effects of Printex 90 on osteogenic differentiation of MSCs.

### Printex 90 resulted in mitochondrial dysfunction during osteogenesis

Mitochondria are ubiquitous in eukaryotic cells and they are best known for ATP production. Mitochondria are also involved in other cellular activities such as differentiation, aging and cell death [45]. Numerous studies have demonstrated that mitochondria play critical roles in osteogenesis of MSCs [45, 46]. Due to their structures and functions, mitochondria are potentially susceptible to xenobiotics such as nanomaterials. Therefore, we hypothesized that Printex 90 may cause mitochondria damage and thus affect osteogenesis.

**Fig. 5:**
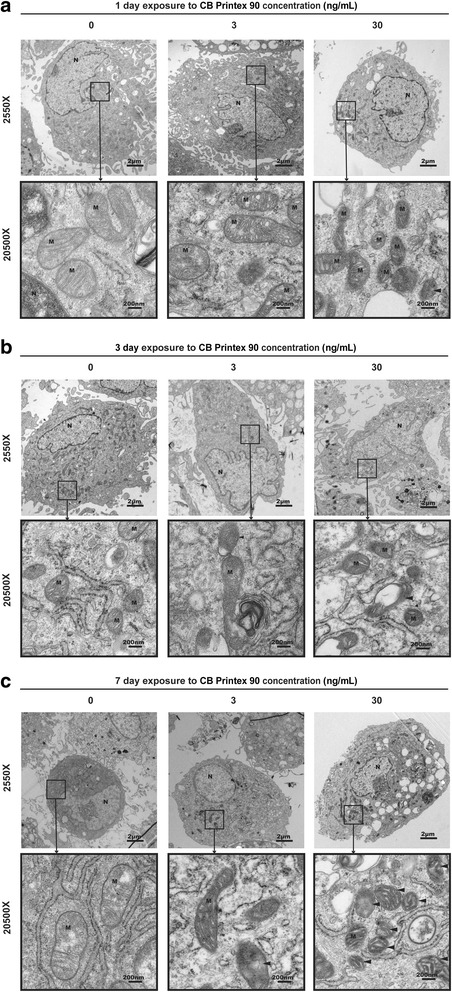
Effect of Printex 90 on mitochondrial morphology of MSCs during osteogenesis. MSCs exposed to 3 ng/mL and 30 ng/mL Printex 90 were induced to osteogenic differentiation for 1 d, 3 d and 7d. Representative low-magnification (2550×) and high-magnification (20,500×) TEM images showed that although mitochondria structure had few dissolved cristae (black arrow) when exposed to 30 ng/mL CB for 1 d (**a**), the gradual loss of integrity of the cristae structure and vacuolar degeneration (black arrows) was found when compared to the mitochondria in 0 ng/mL Printex 90 at 3 d (**b**). Printex 90 had more severe damage to mitochondria showing dissolved mitochondrial cristae (black arrows) in 3 ng/mL CB treated cells and the swelling and abnormal density of mitochondria (black arrows) in 30 ng/mL at 7 d(**c**). Scale bar = 2 μm (highlight area = 200 nm). N: nucleus; M: mitochondria

CB nanoparticles can be attracted by protons in the mitochondrial compartment perturbing the mitochondrial membrane in polar protein regions and decreasing mitochondrial membrane potential and permeability [47]. Therefore the morphological changes of mitochondria could reflect the direct impairment resulting from nanoparticle exposure. We conducted a series of dose-effect TEM studies to evaluate the changes of mitochondria of MSCs by Printex 90-induced toxicity during osteogenesis within a 7 d exposure period. A gradual loss of integrity of the cristae structure and vacuolar degeneration followed the extended exposure time (Fig. 5a-b). Dissolved mitochondrial cristae, swelling and abnormal density of mitochondria in cultured MSCs were observed at 7 d exposure to 30 ng/mL Printex 90 (Fig. 5c).

**Fig. 6:**
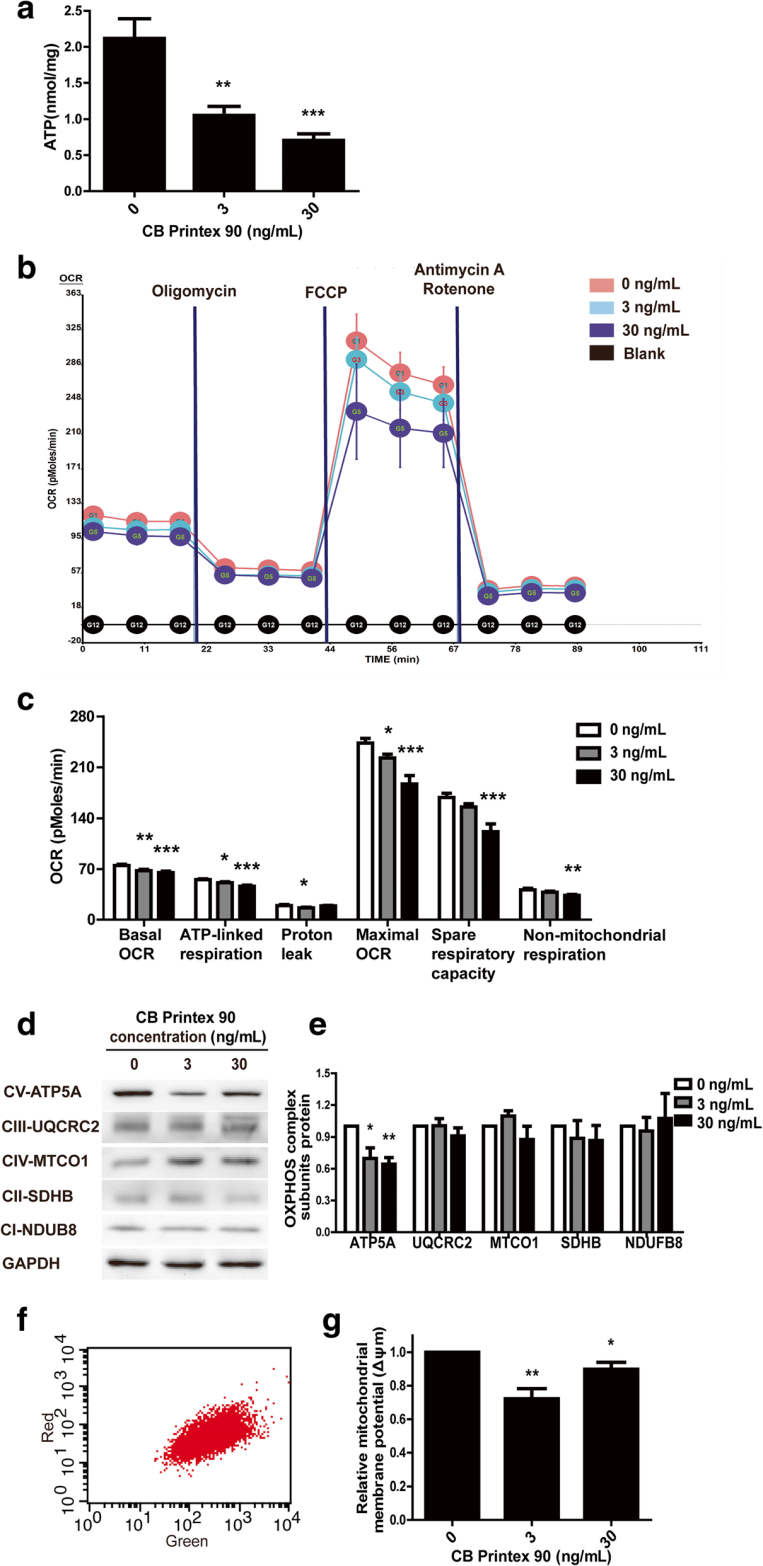
Effects of Printex 90 on mitochondrial functions in the process of MSCs osteogenic differentiation. MSCs were treated with 3 ng/mL and 30 ng/mL Printex 90 and induced to osteogenic differentiation within 7 d. **a** Reduced ATP content was found in CB Printex 90-treated cells when compared with control after osteo-induction for 7 d. **b-c** XF-96 Flux Analyzer showed the decreased basal oxygen consumption rate (OCR), ATP-linked respiration, proton leak, the maximal OCR, spare respiratory capacity (SRC) and non-mitochondrial respiration after osteo-induction for 4 d. **d** Western blotting showed the protein expression of five mitochondrial oxidative phosphorylation (OXPHOS) complex subunits after osteo-induction for 7 d. **e** Quantification of the Western blots. **f** Representative image from flow cytometer with cells stained by JC-10 probe after osteo-induction for 7 d. **g** The relative red/green ratio of cells stained by JC-10 probe

ATP content and cellular respiration are important indicators for normal mitochondrial function [48]. The majority of ATP is produced via OXPHOS of the mitochondrial respiratory chain driven by the transmembrane electrical potential [49]. We compared the ATP production in Printex 90-treated groups with control during osteogenesis. In control cells the ATP content was 2.1 ± 0.3 nmol/mg protein while the 3 ng/mL and 30 ng/mL Printex 90 treatments decreased ATP production to 1.1 ± 0.1 and 0.7 ± 0.1 nmol/mg protein, respectively (Fig. 6a). This suggested that Printex 90 treatment caused OXPHOS damage.

ATP synthesis is coupled with oxygen consumption because the electron transport chain (ETC) in the inner membrane of mitochondria transfers electrons to oxygen resulting in ATP generation. Reduction of ATP by Printex 90 treatment led us to evaluate the oxygen consumption of osteogenic differentiated MSCs using a mitochondrial stress assay. We optimized the cell number and the concentration of each chemical used in the experiments (data not shown) and the optimized conditions were used (1 μM oligomycin, 1.8 μM FCCP, 0.5 μM rotenone and 0.5 μM Antimycin A). We found accumulated damages to mitochondrial functions in the process of MSCs osteogenic differentiation (Additional file 4: Figure S3). Figure 6b and c, show that both 3 ng/mL and 30 ng/mL Printex 90 produced significant declines in basal OCR. These were 67.5 ± 1.8 and 65.1 ± 1.7 pmol/min respectively, compared to the control (74.8 ± 1.7 pmol/min). The basal OCR is composed of respiration linked to ATP production and proton leakage. ATP-linked respiration (Basal OCR - Oligomycin response) was significantly lower (*p* < 0.05) in both 3 ng/mL and 30 ng/mL Printex 90 treated groups. These were 50.9 ± 1.5 and 46.2 ± 1.7 pmol/min respectively, compared with control (55.2 ± 1.2 pmol/min), which was consistent with the decreased ATP production demonstrated in Fig. 6a. Proton leak (Oligomycin response – Antimycin A & Rotenone response) in 3 ng/mL treated cells was significantly lower (*p* < 0.05) than control, 16.2 ± 0.9 vs.19.6 ± 1.2 pmol/min, but in 30 ng/mL Printex 90 treated cells, it was similar to the normal level (19.0 ± 0.8 pmol/min). The non-increase in electron leakage was consistent with our data that the reactive oxygen species (ROS) generation was similar between the control and treatment groups (data not shown). After injection of FCCP which ablates mitochondrial membrane potential, both 3 ng/mL and 30 ng/mL Printex 90 treatments significantly suppressed the maximal OCR (Fig. 6b-c), 222.7 ± 5.2 and 186.7 ± 11.8 pmol/min, respectively. This suggested either inefficiency in substrate supply and oxidation or decreased activity of the enzymes responsible for synthesizing ATP. The spare respiratory capacity (SRC) (Maximal OCR - Basal OCR) and non-mitochondrial respiration (after injection of Antimycin A & Rotenone) were not significantly different between control and 3 ng/mL Printex 90-treated cells while 30 ng/mL Printex 90 suppressed (*p* < 0.05) the SRC and non-mitochondrial respiration (Fig. 6b-c).

We determined if the decrease in maximal OCR could be attributed to the modulation of individual components of the ETC, which is composed of five multi-enzymatic complexes (CI-CV) in the inner mitochondrial membrane. Western blotting showed that 3 ng/mL and 30 ng/mL Printex 90 decreased the protein expression of CV (ATP5A, ATP synthase, alpha subunit) up to 70% and 64% that of the control, but the protein expression of the other four ETC enzymatic complexes was not significantly altered (Fig. 6d-e). The suppression of CV in Printex 90-treated cells could partially explain the decreased maximal OCR seen in Fig. 6b-c.

The reduced activity of the respiratory chain in Printex 90-treated cells led us to analyze the mitochondrial membrane potential (ΔΨ_m_), a driving force for electron flow and ATP production. A JC-10 fluorescent probe was used since JC-10 can be transformed from a red aggregation to green monomer when the mitochondrial membrane potential is reduced. The ratio of red to green fluorescence of JC-10 is dependent only on membrane potential. Figure 6f shows a representative fluorescence intensity histogram. The red/green ratio in 3 ng/mL treated groups was significantly lower (*p* < 0.05) (72 ± 6%) than the control (Fig. 6g). The mitochondrial membrane potential in 30 ng/mL Printex 90-treated cells was similar to, but still lower than, the control (Fig. 6g), indicating Printex 90-induced depolarization of mitochondria.

### Effects of Printex 90 on mitochondrial biogenesis, dynamics and mitophagy during osteogenesis

The mitochondrial dysfunctions stimulated investigation of the degree to which mitochondrial biogenetics, mitochondrial fusion and fission (mitochondrial dynamics) or mitophagy, were affected in Printex 90-treated cells during osteogenesis. Mitochondrial biogenesis, dynamics and mitophagy are, as a group, responsible for mitochondrial quantity and quality control [18]. However, under stress and mitochondrial damage, mitophagy is initiated. Mitophagy is primarily regulated by the PINK1/Parkin pathway. Once on the mitochondria, Parkin could degrade mitochondrial proteins, including Mfn1/2 which is crucial to fusion, Drp1, which is involved in fission, and PARIS (ZFN746) which is an inhibitor of PGC1α. These actions demonstrate the interactions of mitochondrial biogenesis, dynamics, and mitophagy [50].

**Fig. 7:**
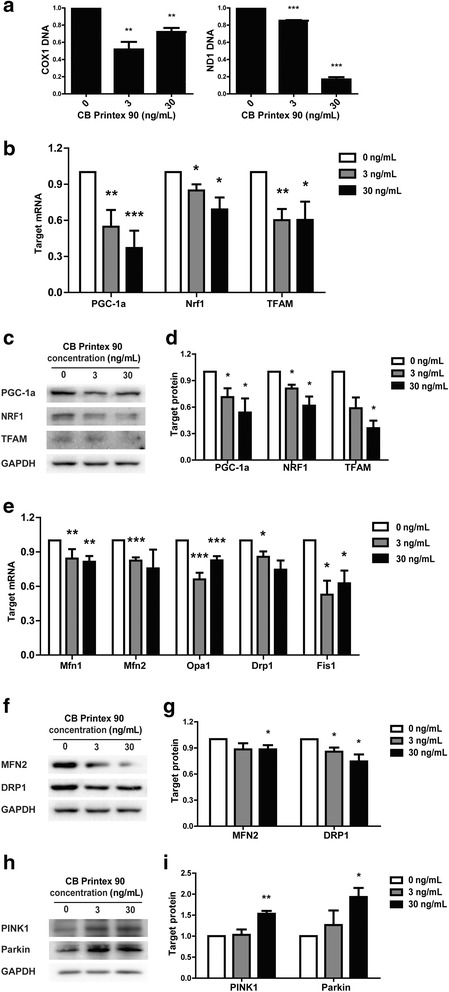
Impairment of mitochondrial biogenesis, dynamics and mitophagy. MSCs were treated with 3 ng/mL and 30 ng/mL Printex 90 and induced to osteogenic differentiation for 7 d. **a** RT-PCR demonstrated the lower expression of mtDNA, including COX1 and ND1 after osteo-induction for 7 d. After treatment for 7 d, mRNA or proteins were isolated for RT-PCR (**b**)&(**e**) and Western blot (**c**)&(**f**)&(**h**). (**d**)&(**g**)&(**i**) The quantification of Western blots

Mitochondria have their own DNA genome (mtDNA) that encodes 13 essential protein subunits of the respiratory chain. The ratio of mtDNA vs. nuclear DNA (ntDNA) is commonly used to reflect the mtDNA content and mitochondrial biogenesis [51]. ND1 and COX1, two of the 13 genes encoded by mtDNA, were selected for this study. The ratios of both ND1/ntDNA and COX1/ntDNA were significantly reduced in Printex 90-treated groups compared to the control (Fig. 7a), demonstrating the inhibitory effects of Printex 90 on mtDNA biogenesis. The replication, transcription and translation of mtDNA are accurately controlled by the PGC-1α, Nrf1/Nrf2, and TFAM [21]. Therefore, we evaluated the gene expression and proteins of these biogenesis related factors to determine if the decrease in mtDNA copy number of Printex 90-treated cells was caused by impaired biogenesis. Using real-time PCR and Western Blotting, we found that TFAM, PGC-1α and Nrf1 were dramatically decreased at both the mRNA and the protein level (*p* < 0.05, Fig. 7b-d), confirming the inhibitory effects of Printex 90 on mitochondrial biogenesis.

Mitochondria constantly undergo fusion and fission to adapt to cellular energetic demands and to maintain their proper functions. We assessed the gene expression and proteins of fusion and fission related factors, including Mfn1, Mfn2, Opa1, Drp1 and Fis1. RT-PCR demonstrated that Mfn1, Mfn2, Opa1, Drp1 and Fis1 were dramatically decreased at the mRNA level (Fig. 7e). Mfn2 and Drp1 were studied using Western blot and they were also suppressed at the protein level in Printex 90-treated cells (*p* < 0.05, Fig. 7f-g). Together with RT-PCR data, we confirmed that mitochondrial dynamics, including both fusion and fission, were damaged by Printex 90 treatment during osteogenesis.

Mitophagy is an important mitochondrial quality control mechanisms. It operates through the degradation of dysfunctional mitochondria, which is primarily regulated by the PINK1/Parkin pathway. PINK1 is primarily located in the mitochondrial inner membrane, while Parkin is primarily found in the cytoplasm. When mitochondrial damage occurs, PINK1 accumulates on the outer membrane of the mitochondria where it recruits Parkin into the mitochondria and initiates the mitophagy process [52]. We assessed the protein expression of Parkin in mitochondria and PINK1 at the total cell level to evaluate whether mitophagy was altered by Printex 90 treatment. Western blotting demonstrated that both PINK1 and mitochondrial Parkin were significantly increased in Printex 90-treated osteogenic differentiated cells (Fig. 7h-i). Several potential substrates of Parkin have been reported, including Mfn1, Mfn2, and Drp1 [53, 54]. Therefore, the suppressed Mfn1, Mfn2 and Drp1 we observed could be attributed to the elevated Parkin. Furthermore, the increase in mitophagy reflected that the mitochondria were damaged, which was also supported by our mitochondrial function analysis indicating decreased ATP, depolarization of mitochondria, and respiration abnormities (Fig. 6).

Because the BMP-pathway and the canonical WNT-pathway are likely involved in osteogenic differentiation, we screened multiple signal molecules on the BMP and WNT pathways during MSC differentiation. The expression levels of Bmp2 and Bmp4 in the BMP-pathway and Wnt3a and Wnt4 in the WNT-pathway were significantly inhibited during the Printex 90 interrupted osteo-induction in a time and dose dependent manner (Additional file 5: Figure S4). Mitochondrial biogenesis can be upregulated by Wnt and BMP signaling and this upregulation contributes to the osteoblastic differentiation of MSC cell lines [55]. Our results show a logical relationship from molecular level to the subcellular organelles and the mitochondria defects.

**Fig. 8:**
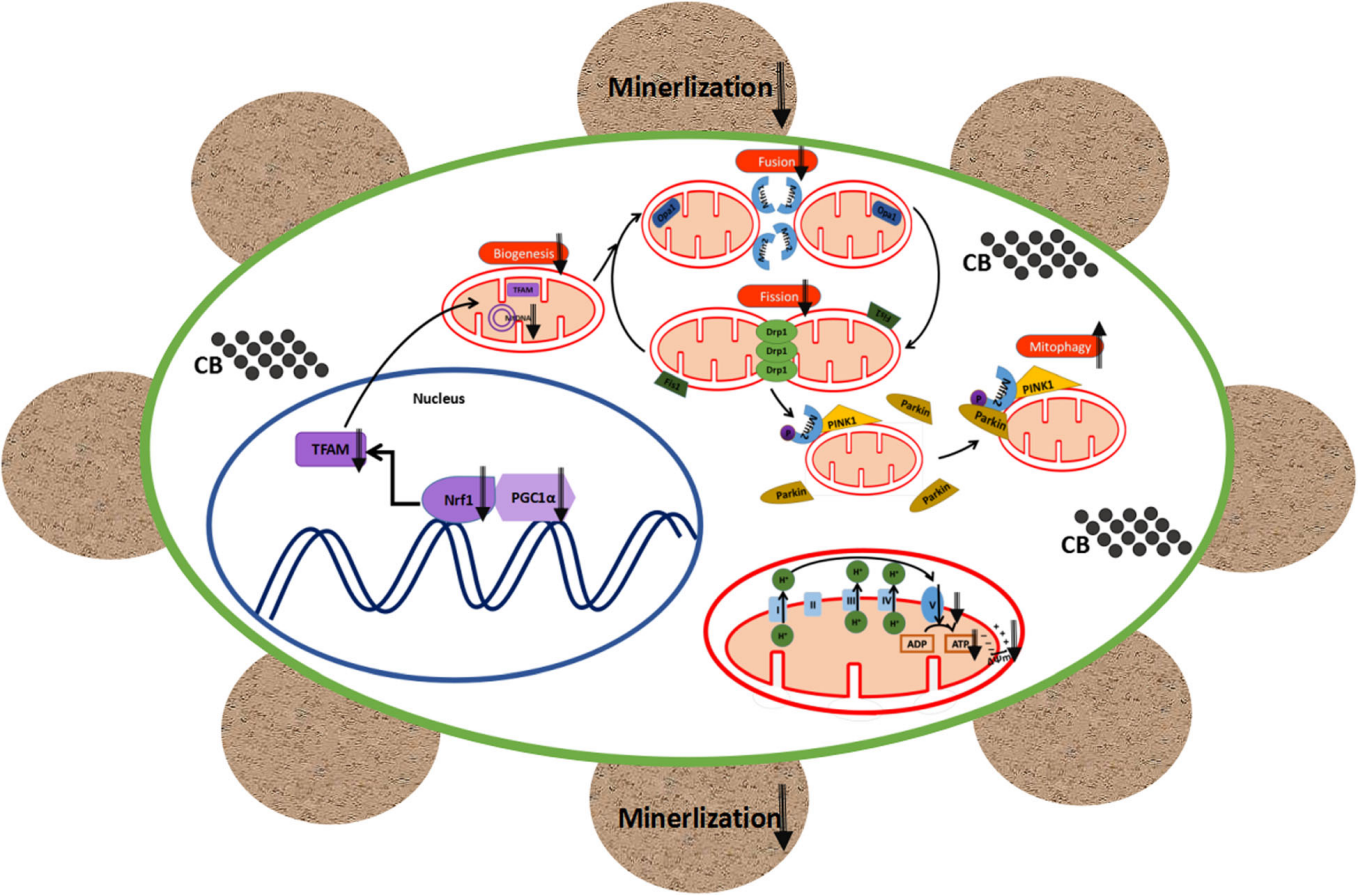
A proposed model that nano-sized CB particles (Printex 90) resulted in dysfunctional mitochondria and thus inhibited osteogenesis of MSCs. Upon Printex 90 treatment, PGC1α, Nrf1 and TFAM were down-regulated followed by the relative lower copy number of mtDNA, indicating decreased mitochondrial biogenesis. Mitochondria dynamics including fusion and fission were also damaged as demonstrated by the suppressed expression of dynamics related factors, including Mfn1, Mfn2, Opa1, Drp1 and Fis1. Additionally, the accumulation of Parkin in mitochondria and PINK1 in total cell demonstrated increased mitophagy. In the meantime, mitochondrial functions were impaired, including decreased ATP production, depolarization of mitochondria and abnormal respiration. Therefore, the osteogenesis of MSCs was inhibited, demonstrated by the down-regulation of Runx2, Alp and B glap, and the poor mineralization. Collectively, CB Printex 90 could induce mitochondrial dysfunction and result in increased mitophagy and suppressed mitochondrial biogenesis and dynamics. Ultimately, the osteogenesis of MSCs was inhibited

Mitochondria are potential target organelles for toxicity caused by nanomaterials. Numerous nanoparticles (e.g., Ag nanoparticles, carbon nanotubes, TiO_2_ nanoparticles, silica nanoparticles, zinc oxide nanoparticles, quantum dots and fullerenes) can result in mitochondrial dysfunction, by the alteration of respiration, OXPHOS, mitochondrial permeability, mitochondrial membrane permeability transition (MPT) or generation of ROS [42, 56–58]. Impaired mitochondrial biogenesis, dynamics and mitophagy by nanoparticles have also been reported [57–61]. Alterations of mitochondria can play indispensable roles in the osteogenic differentiation of MSCs [46, 48]. We demonstrated that Printex 90 could induce mitochondrial dysfunction during the osteogenic differentiation of MSCs(Fig. 8). This was caused by the reduction of ATP level and oxygen consumption, the decreased expression of OXPHOS subunit and reduced mitochondrial membrane potential. Mitochondrial damage resulted in increased mitophagy and concomitantly suppressed mitochondrial biogenesis and dynamics. Ultimately, the osteogenesis of MSCs was inhibited (Fig. 8). Studies have also shown that exposure to other nanoscale materials can be associated with blood mitochondrial abundance and mitochondrial alteration occurring at an early stage [62, 63]. These data suggest that specific mitochondrial markers may be useful biomarkers for toxicity assessments of nanomaterials.

Early changes in mitochondrial morphology and alterations in the bioenergetic profile are important for osteogenesis of MSCs, since the upregulation of mitochondrial biogenesis are the hallmarks of MSCs differentiation [24, 25, 64]. Many mechanisms can regulate the differentiation and mitochondria function of MSCs. For example, ROS-dependent phosphorylation of FOXO3 at serine 294, which is mediated by MAPK8 kinase, is required to induce autophagy and reduce elevated ROS levels resulting from the increased mitochondrial respiration during osteoblast differentiation [64]. Post-translational protein modifications in the regulation of mitochondrial function such as phosphorylation, glycosylation and acetylation could favor osteogenic differentiation and play a critical role in the lineage commitment of MSCs.

## Conclusions

This is the first study demonstrating that Printex 90 inhibition of the osteogenic differentiation of MSCs is associated with mitochondrial dysfunction and affects regulators of mitochondrial biogenesis, dynamics, and mitophagy (Fig. 8). Additional study will be required for clarifying the mechanisms, but our work indicates that regulators of mitochondrial biogenesis, dynamics and mitophagy could be potential biomarkers for Printex 90-induced toxicity. Our findings have public health implications for developing regulatory strategies to reduce Printex 90 exposure and mitigate its harmful effects.

## Additional files

Additional file 1: **Table S1.** Primers used for real-time polymerase chain reaction (DOC 37 kb)

Additional file 2: **Figure S1.** Characterization of Printex 90. (a) Summarization of the size and zeta potential of CB dispersed in PBS, H_2_O and the complete culture medium. (b) Particle-size distribution of CB prepared in PBS, H_2_O and the culture medium. (JPEG 2194 kb)

Additional file 3: **Figure S2.** Absorbance of the supernatant of the CCK-8 reagent diluted with the cultured medium containing 0 to 30 μg / mL Printex 90 at 450 nm. After two hr. incubation at 37 °C, the solution was centrifuged by 1500 g for 2 min, and the absorbance of supernatant was measured immediately by Infinite M200 Pro (TECAN, Switzerland) at 450 nm, four repeats at each dosage have been calculated. (TIFF 12726 kb)

Additional file 4: **Figure S3.** The protein content of oxygen consumption rate (OCR) (a), and OCR values of different dose groups normalized by the protein content at Day 1 (b), and the protein content of oxygen consumption rate (OCR) (c), and OCR values of different dose groups normalized by the protein content at 3 d (d). Decreased proton leak was showed by XF-96 Flux Analyzer after 1 d osteo-induction (a), whereas other parameters including basal oxygen consumption rate (OCR), ATP-linked respiration, proton leak, the maximal OCR as well as non-mitochondrial respiration were inhibited after 3 d osteo-induction (c). No significant shifts for the values normalized by the protein content were found when compared to the values calculated without protein normalization (also see Fig. 6b-c). (JPEG 5670 kb)

Additional file 5: **Figure S4.** The effect of Printex 90 on the expression of BMP-pathway and WNT-pathway molecules during osteogenesis of MSCs. MSCs were treated with 3 ng/mL and 30 ng/mL CB and induced to osteo-differentiation for 7 d. RT-PCR demonstrated the expression of BMP-pathway genes, Bmp2 and Bmp4 (a), and WNT-pathway genes, Wnt3a and Wnt4 (b). (JPEG 2271 kb)

## Abbreviations

ALP: Alkaline phosphatase
ATP: Adenosine 5′-triphosphate
Bglap (Ocn): Osteocalcin
Bmp2: Bone morphogenetic protein 2
Bmp4: Bone morphogenetic protein 4
CB: Carbon black
CCK-8: Cell-Counting Kit 8
ETC: Electron transport chain
FCCP: Carbonyl cyanide 4-(trifluoromethoxy) phenylhydrazone
GAPDH: Glyceraldehyde-3-phosphate dehydrogenase
Mfn: mitofusin
MPT: Mitochondrial membrane permeability transition
MSCs: Mesenchymal stem cells
mtDNA: Mitochondrial DNA
Nrf: Nuclear respiratory factor
ntDNA: Nuclear DNA
OCR: Oxygen consumption rate
Opa1: Optic atrophy protein 1
PGC-1α: Peroxisome proliferator-activated receptor-*γ* co-activator 1 alpha
ROS: Reactive oxygen species
Runx2: Runt-related transcription factor 2
SRC: Spare respiratory capacity
TEM: Transmission electron microscopy
TFAM: Mitochondrial transcription factor A
Wnt3a: Wnt family member 3A
Wnt4: Wnt family member 4
