# Supplementary material for: Carbon black suppresses the osteogenesis of mesenchymal stem cells: the role of mitochondria
Source: Part Fibre Toxicol. 2018 Apr 12;15:16. doi: 10.1186/s12989-018-0253-5 (PMC5897950; doi:10.1186/s12989-018-0253-5)
Supplement: Supplementary file 1 — Table S1. Primers used for real-time polymerase chain reaction (DOC 37 kb) [file 12989_2018_253_MOESM1_ESM.doc]

| Gene | Primer-F(5'-3') | Primer-R(5'-3') |
| --- | --- | --- |
| GAPDH | GGCACAGTCAAGGCTGAGAATG | ATGGTGGTGAAGACGCCAGTA |
| ALP | GGGACTGGTACTCGGACAAT | GGCCTTCTCATCCAGTTCAT |
| Bglap | CATGAGGACCCTCTCTCTGC | TGGACATGAAGGCTTTGTCA |
| Runx2 | GCACCCAGCCCATAATAGA | TTGGAGCAAGGAGAACCC |
| COX1 | TAATTCGAGCTGAACTAGGAC | TACAAGTCAGTTCCCGAAGC |
| ND1 | GTAATTGCGTAAGACTTAAAACC | CCTAGAAATAAGAGGATTTAAACC |
| PGC-1a | ACATGTGCAGCCAAGACTCTGTA | ACACCACTTCAATCCACCCAGAA |
| Nrf1 | GAGTGACCCAAACCGAACA | GGAGTTGAGTATGTCCGAGT |
| TFAM | TTTCGTGGCTGTGTGTAGGC | TCTTAGCACGCCCCACATTC |
| Mfn1 | TCATAGTTGTTGGGGGCGTGAT | TCGCACGAGTAGTCCAAGTCAG |
| Mfn2 | CTGGTTGGACAGCGAACTCAAC | ACAAGACAGTGGGTGCTTTCCT |
| Opa1 | CAGCTGGCAGAAGATCTCAAG | CATGAGCAGGATTTTGACACC |
| Drp1 | GCAAGAGAACTACCTTCCGCTG | GTTGTCGGTTCCTGACCACCAT |
| Fis1 | CTGGTAGGCATGGCCATTGTTG | GATGGACAGCCCCTCTTTCAGT |
| BMP2 | ACGTCCTCAGCGAGTTTGAGTT | GGCGGTACAGGTCGAGCATA |
| BMP4 | GAGCGCCATTTCCATGTTGT | CTCCGGACTGCCTGATTTCA |
| Wnt3a | TCATGAACTTGCACAACAATGA | CCGTCTTAAACTGGTCATAGCC |
| Wnt4 | GCCACGCACTAAAGGAGAAG | GGCCTTAGACGTCTTGTTGC |
|  |  |  |

Table S1.Primers used for real-time polymerase chain reaction
