# Supplementary figures and images for: Carbon black suppresses the osteogenesis of mesenchymal stem cells: the role of mitochondria
Source: Part Fibre Toxicol. 2018 Apr 12;15:16. doi: 10.1186/s12989-018-0253-5 (PMC5897950; doi:10.1186/s12989-018-0253-5)

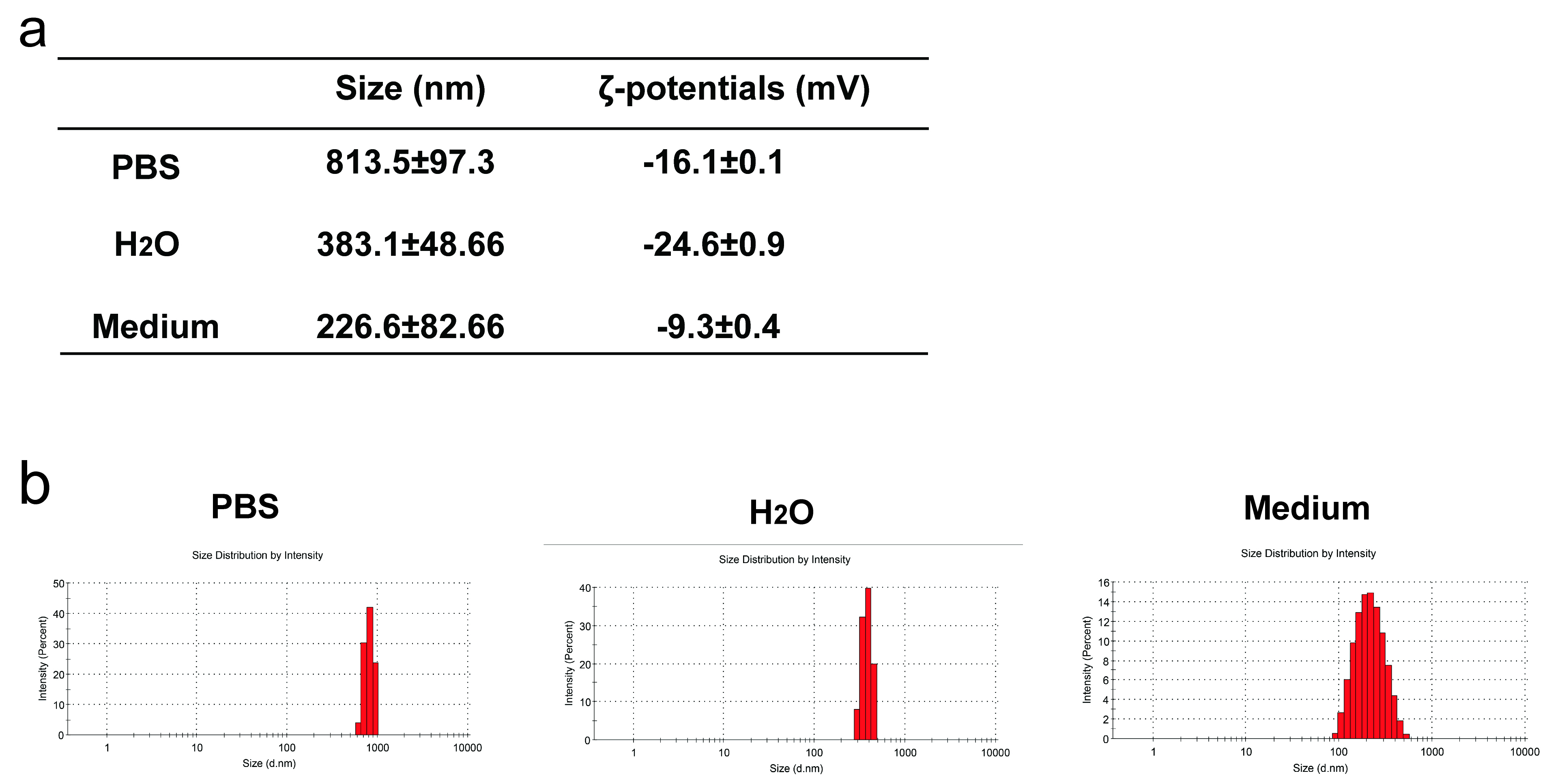

Supplement: Supplementary file 2 — Figure S1. Characterization of Printex 90. (a) Summarization of the size and zeta potential of CB dispersed in PBS, H2O and the complete culture medium. (b) Particle-size distribution of CB prepared in PBS, H2O and the culture medium. (JPEG 2194 kb) [file 12989_2018_253_MOESM2_ESM.jpg]

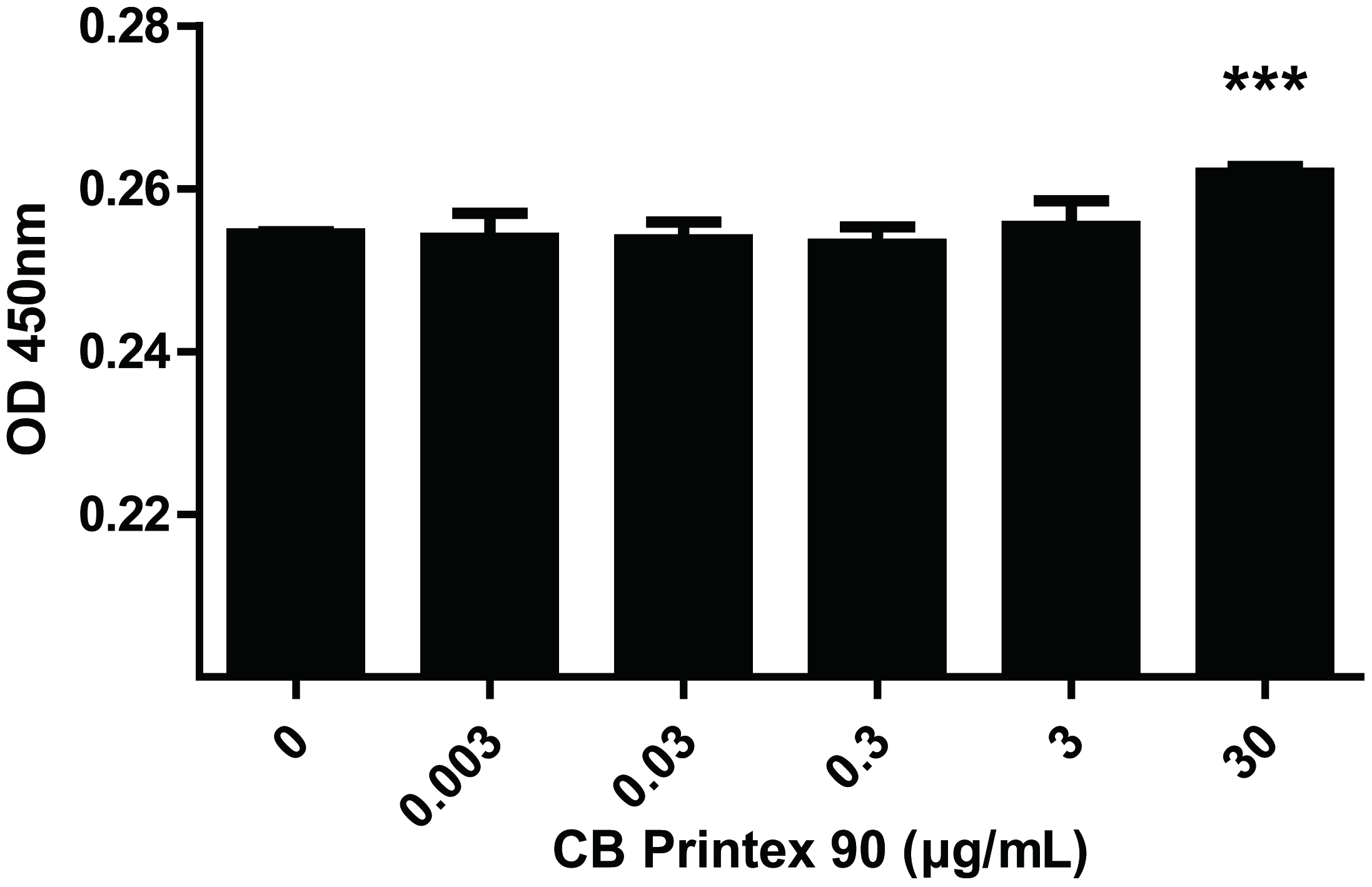

Supplement: Supplementary file 3 — Figure S2. Absorbance of the supernatant of the CCK-8 reagent diluted with the cultured medium containing 0 to 30 μg / mL Printex 90 at 450 nm. After two hr. incubation at 37 °C, the solution was centrifuged by 1500 g for 2 min, and the absorbance of supernatant was measured immediately by Infinite M200 Pro (TECAN, Switzerland) at 450 nm, four repeats at each dosage have been calculated. (TIFF 12726 kb) [file 12989_2018_253_MOESM3_ESM.tif]

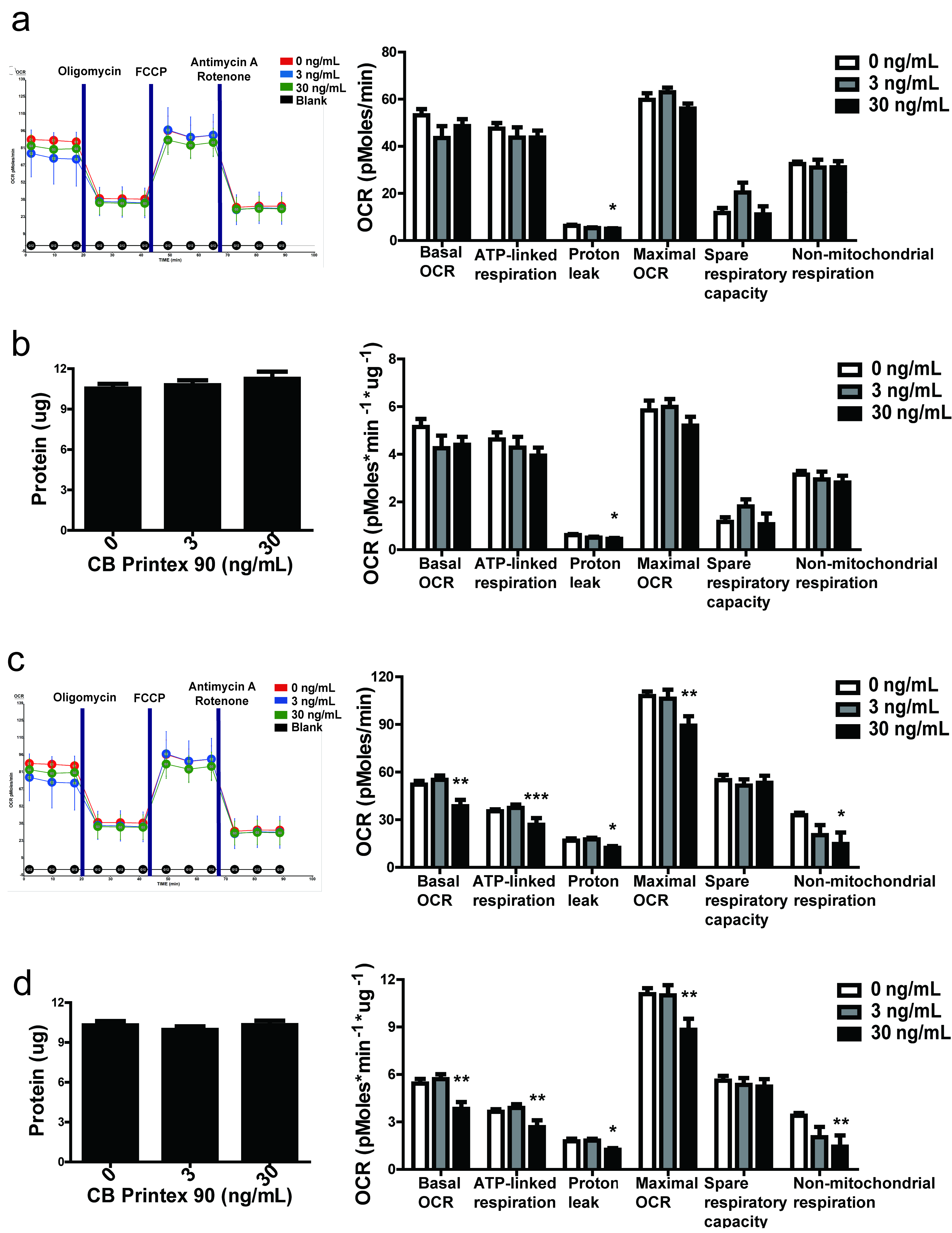

Supplement: Supplementary file 4 — Figure S3. The protein content of oxygen consumption rate (OCR) (a), and OCR values of different dose groups normalized by the protein content at Day 1 (b), and the protein content of oxygen consumption rate (OCR) (c), and OCR values of different dose groups normalized by the protein content at 3 d (d). Decreased proton leak was showed by XF-96 Flux Analyzer after 1 d osteo-induction (a), whereas other parameters including basal oxygen consumption rate (OCR), ATP-linked respiration, proton leak, the maximal OCR as well as non-mitochondrial respiration were inhibited after 3 d osteo-induction (c). No significant shifts for the values normalized by the protein content were found when compared to the values calculated without protein normalization (also see Fig. 6b-c). (JPEG 5670 kb) [file 12989_2018_253_MOESM4_ESM.jpg]

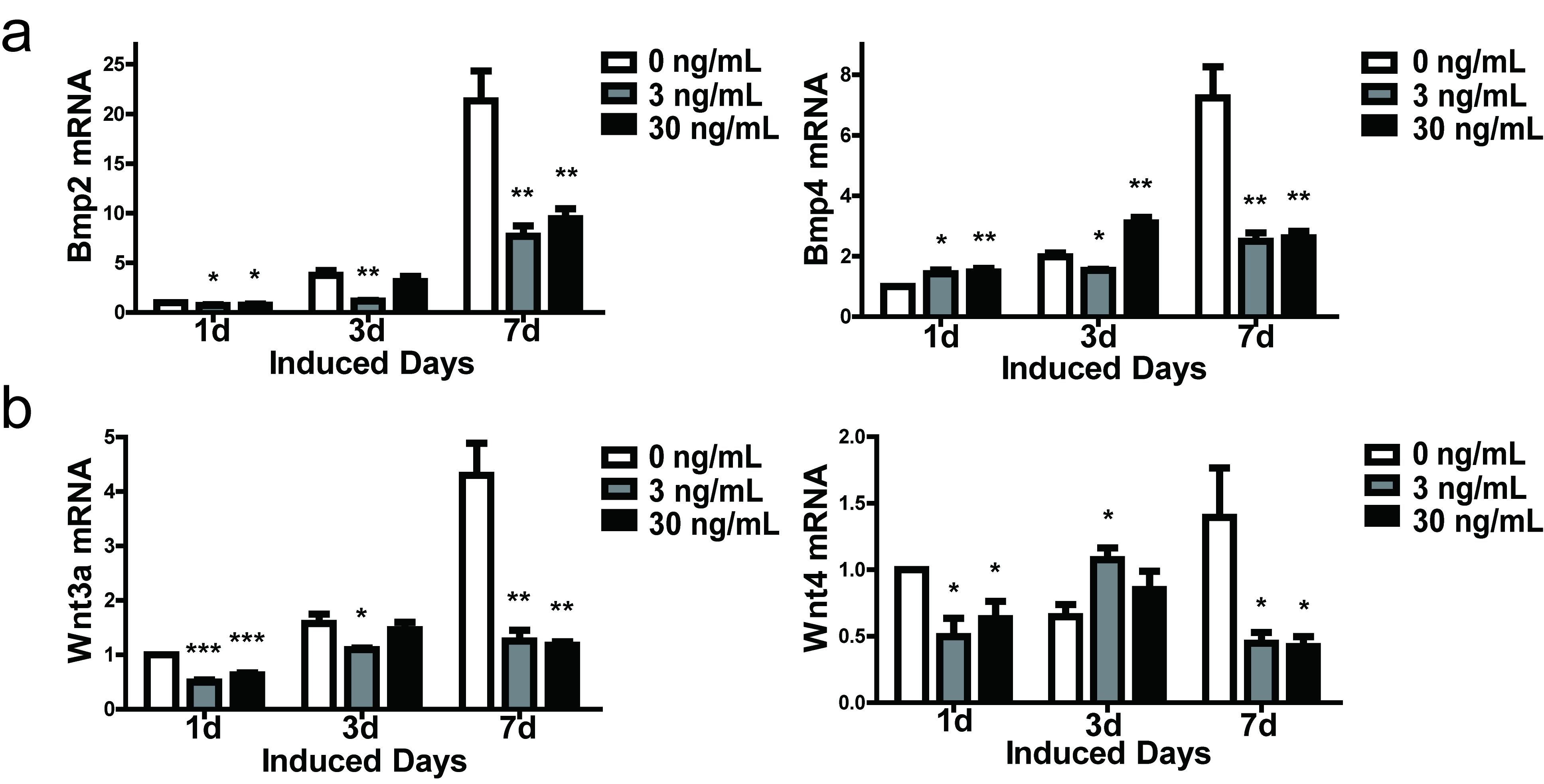

Supplement: Supplementary file 5 — Figure S4. The effect of Printex 90 on the expression of BMP-pathway and WNT-pathway molecules during osteogenesis of MSCs. MSCs were treated with 3 ng/mL and 30 ng/mL CB and induced to osteo-differentiation for 7 d. RT-PCR demonstrated the expression of BMP-pathway genes, Bmp2 and Bmp4 (a), and WNT-pathway genes, Wnt3a and Wnt4 (b). (JPEG 2271 kb) [file 12989_2018_253_MOESM5_ESM.jpg]
